# Online flipped classroom with team-based learning promoted learning activity in a clinical laboratory immunology class: response to the COVID-19 pandemic

**DOI:** 10.1186/s12909-022-03917-3

**Published:** 2022-12-03

**Authors:** Yonghui Feng, Bin Zhao, Jun Zheng, Yajing Fu, Yongjun Jiang

**Affiliations:** Department of Clinical Immunology, The First Hospital of China Medical University, NO.155 Nanjing North Street, Heping District, Shenyang, Liaoning China

**Keywords:** Clinical immunology, COVID-19, Learning activity, Flipped classroom, Team-based learning

## Abstract

**Background:**

Given the rapid development of clinical immunology technologies, students majoring in laboratory medicine should master the technological principles and application of clinical laboratory immunology. However, many are required to take online courses due to COVID-19 restrictions, which highlights the need to revisit teaching strategies. Recently, various medical education courses (such as Biochemistry, Physiology, etc.) have implemented the flipped classroom (FC) and team-based learning (TBL) methods, resulting in more positive teaching evaluations. To promote the students' mastery of the difficult knowledge effectively during the online teaching work, we evaluated the performance of online FC-TBL in a clinical laboratory immunology course.

**Methods:**

Sixty-two third-year students from two classes majoring in Laboratory Medicine were recruited and divided into two groups, including one group with traditional lecture-based learning teaching strategy (LBL group) and the other group with LBL or online FC combined with TBL teaching strategy (FC-TBL group). We selected three chapters to conduct FC-TBL teaching in class. All participants took in-class quizzes and final examinations that targeted the same knowledge points. Finally, all participants completed anonymous questionnaires asking for their perceptions of the respective teaching models. In addition, we conducted a survey of teaching suggestions by a FC-TBL class of students majoring in Laboratory Medicine.

**Results:**

The FC-TBL group (vs LBL group) had significantly higher scores on the in-class quizzes and final examinations, and also reported high satisfaction with the FC-TBL model. These findings indicate that FC-TBL is suitable for clinical laboratory immunology, as the participants quickly gained essential knowledge. Specifically, FC-TBL helped to “increase learning motivation,” “promote self-directed learning skills,” “extend more related knowledge,” “enhance problem-solving abilities,” “enhance clinical reasoning abilities,” and “enhance communication skills.” For participants’ suggestions, 48.38% (15/31) students held positive attitude to FC-TBL teaching strategy compared to 25.81% (8/31) students who considered FC-TBL teaching strategy still needs continuous improvement, and 25.81% (8/31) students reported that they believed FC-TBL teaching strategy was perfect and no further suggestions.

**Conclusions:**

Online FC-TBL effectively enhanced learning activity among students of a clinical laboratory immunology course. This is particularly useful in the COVID-19 context.

## Background

As a critical course offering, clinical laboratory immunology instills medical students with strong professionalism and practical knowledge, which is increasingly important due to the rapid expansion of clinical immunology technologies, many of which play pivotal roles in the diagnosis and management of abnormal immune function [1]. At universities in China, clinical laboratory immunology is designed to bridge basic and clinical medical knowledge. Students are thus expected to gain a thorough understanding of the technological principles and application of clinical laboratory immunology, which is essential for making clinical diagnoses on issues such as autoimmune disease, cancer, and infectious disease [2]. However, there is currently no specific method for evaluating the various approaches to teaching clinical laboratory immunology in the medical laboratory setting. Especially given the increasing popularity of new teaching strategies, it is therefore important to explore evaluation procedures, with the aim of both enhancing interest among students and ensuring that they are provided with quality medical education.

Given the rapid development of modern clinical immunology theories and applied technologies such as Trained immunity, Immunophotonics, Flow Cytometry and Immunofluorescence techniques, it is often difficult to implement traditional teaching strategies in medical education, as many do not allow for effective student–teacher interactions, especially with limited teaching hours [3, 4]. Under these conditions, students passively accept knowledge through the lecture-based learning (LBL) method, which has shown to be ineffective and unresponsive [5].

Flipped classroom (FC) and team-based learning (TBL) teaching strategies mainly derived from student-centered learning theories, based on the theories of Piaget and Vygotsky [6]. As one of the active learning approaches, the flipped classroom requires students to play an active role in class and construct information by themselves [7]. Students gain knowledge points before class, thus enabling teachers and students to “flip” roles during class [8, 9]. In this context, students are better able to discuss course subjects both inside and outside the classroom, including with their peers. The classroom roles are flipped so that students become the leading role of learning, while teachers become designers and guides of the learning process [10, 11]. Moreover, teachers can replace the lecture-based approach with a variety of new teaching strategies that increase students’ enthusiasm for learning using active learning strategies including FC while deepening their comprehension of basic knowledges [12]. For example, TBL is another student-centered, teacher-directed cooperative learning method based on Vygotsky’s theory, in which students are affected by own or other team members’ actions in class [13]. More specifically, students are divided into small teams (e.g., five to seven students) prior to class. Typically, they are expected to complete a reading assignment before collaborating as a team to resolve a challenging problem that is assigned by the teacher [14]. During class, students will then hold inter-team discussions while the teacher facilitates, assists, and comments on their proceedings [15]. Hence, TBL teaching strategy has been proven to improve students’ attitude, success and motivational levels [6]. Cooperation with FC and TBL will increase the effectiveness of the teaching–learning process compared to using FC or TBL teaching strategy individually, and shows a positive impact on students’ success, attitudes and behaviors [7, 16, 17].

Although FC and TBL teaching strategies have been used by medical education courses (such as Biochemistry, Physiology, Nursing education, etc.) increasingly, only few studies have focused on these strategies in laboratory medicine courses including clinical laboratory immunology [1, 18–20]. Given the unique advantages of FC and TBL, we believe they can provide enhanced benefits when combined; in clinical laboratory immunology, this approach should help students master relevant principles and applications for use in their future clinical work. As such, this study developed and implemented a FC with TBL (FC-TBL) program for use among students of a clinical laboratory immunology course.

Under the limitations imposed by the coronavirus disease 2019 (COVID-19) pandemic, many students have accepted at-home medical education, which impels teachers to apply online teaching strategies [21]. With the goal of reforming practices to cultivate undergraduate talents and master the difficult knowledges, we relied on our teaching experience to adjust to these major changes by redesigning the teaching strategies used in some chapters of our implemented coursework. As a response to COVID-19 restrictions, we conducted the FC-TBL course online via Tencent Meeting and the Rain Classroom APPs, then made subjective and objective comparisons of the FC-TBL and LBL approaches based on in-class quizzes, examinations, and questionnaires. We thus found that the online FC-TBL (vs LBL) course was more effective for promoting learning interest, team-based problem-solving, and knowledge mastery, which also provide an example and reference for other medicine and life science courses.

## Methods

### Study participants

We recruited 62 third-year students from two classes majoring in Laboratory Medicine at China Medical University for the comparison of two teaching strategies. One class, called LBL group, includes 31 participants exposed to the traditional LBL method prior to COVID-19, and the other class, called FC-TBL group, includes 31 participants that was exposed to the LBL for learning part of clinical laboratory immunology and FC-TBL strategy for learning the remain part of clinical laboratory immunology. As shown in Table 1, the gender distributions were similar, with nearly double the number of females (vs males) in both groups, which were also very similar in age (Table 1). The FC-TBL group was further divided into six teams containing five to six students each. All participants were enrolled in this study with mandatory clinical chemistry, with final exam scores as the basis. In addition, we conducted a survey of teaching suggestions by a FC-TBL class of students majoring in Laboratory Medicine.

**Table 1:** Baseline participant characteristics (*n* = 62)

	LBL group (third-year students)	FC-TBL group (third-year students)	Statistics	*P* value
Total	31	31	-	-
Gender (NO, %)				
Male	10 (32.26)	12 (38.71)	χ^2^ = 0.531	0.600
Female	21 (67.74)	19 (61.29)		
Age (y)	21.19 ± 0.75	21.06 ± 0.77	*t* = 0.668	0.507

### FC-TBL and LBL course structures

The FC-TBL group was provided with the following: Instructors introduced the teaching strategies and corresponding contents to students. We selected three chapters to teach students the online FC-TBL course, while other chapters were conducted with LBL due to the COVID-19 pandemic. The chapters were:

1. Quality Assurance of Clinical Immunoassay (PowerPoint slideshow). The topics were prepared in advance to provide students with a choice. Each team cooperated to systematically present their topic to other students using PowerPoint slides, with an in-class review of the literature.
2. Research Design. Each team designed a study to determine SARS-CoV-2 based immune assays, then displayed relevant PowerPoint slides in class.
3. Cosplay. Students in each team played roles to finish a clinical case with a given disease based on clinical immunology tests. These were accompanied by videos.

In general, the participants were instructed through traditional lecture-based teaching models. Instructors used Rain Classroom APP to deliver pre-class resources, including the assigned reading materials, questions, lectures with PowerPoint slides, and/or videos. All participants were required to read any such materials or questions within a stipulated timeframe. The instructors introduced the FC rules and contents prior to class. They showed one PowerPoint slide on the topic with key points. Then, a student representative from each team presented their cooperation results to the other teams, followed by inter-team discussions under guidance of the instructor during class. Participants who were presenting PowerPoint slides or videos needed to answer questions posed by other teams. The instructors recorded and summarized these questions and answers, which were discussed at the end of class, and issued comprehensive scores based on each team performance. All online FC-TBL classes were conducted via the online Rain Class application (version 4.1) and Tencent Meeting software (version 2.0.0).

By contrast, the LBL group was not provided with preview materials, PowerPoint slides, or videos, and could use only the assigned textbook. These participants accepted teaching contents that were equal to those provided to the FC-TBL group, but their class was teacher-centered and did not include any team-based discussions. Rather, the instructor employed the traditional LBL approach, and clarified any theoretical aspects that were difficult to understand based on their own knowledge, with reference to the course syllabus.

All participants in all groups took the final examination. Following this, we compared the accuracy of students’ answers from the FC-TBL and LBL groups based on their test scores, including those from the in-class quizzes. After revealing all final examination scores, we distributed questionnaires asking each participant to provide their perceptions of the respective teaching models.

### Subjective evaluation methods

**Fig. 1:**
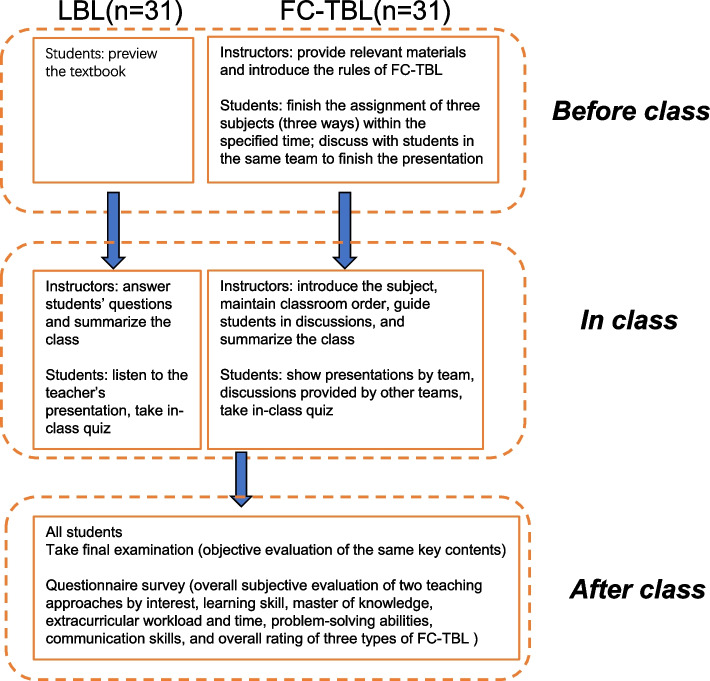
Flowchart showing the study procedure

To assess the level at which participants comprehended each of three course subjects, they completed in-class quizzes and final examinations that targeted the same knowledge points. After completing their final examinations, each participant completed a subjective evaluation questionnaire, which was compiled with referenced to previous research [22]. First, they responded to FC-TBL or LBL by choosing YES or NO on the comparison questionnaire. Thus, separate questionnaire was administered to evaluate their perceptions of the online FC-TBL or LBL, with items rated on a 5-point Likert scale ranging from 1 (strongly disagree) to 5 (strongly agree). They also provided suggestions based on their perceptions. Data collection, calculation and analysis of students’ test results and questionnaires were seriously checked in by two class teachers independently. All students completed the questionnaires anonymously. Figure 1 shows a simplified flowchart of the study procedure.

### Ethical considerations

This study was carried out in accordance to the Declaration of Helsinki. Prior to commencement, we received approval for this study and the curriculum design from both the Department of Teaching Affairs and ethical committee of The First Hospital of China Medical University (2022–344).

### Data analysis

We tested the continuous variables for normality via the Shapiro–Wilk test in this study and conducted an independent samples *t*-test to compare intergroup differences in age as well as clinical chemistry scores. Mann–Whitney *U*-test was used to compare intergroup differences in clinical laboratory immunology scores and accuracy on the in-class quizzes. We used Pearson's chi-squared test to compare the general characteristics of participants as well as participant reactions to the online FC -TBL teaching strategy. Total scores of FC-TBL peer evaluations with five-point Likert scale from each student were calculated and Mann–Whitney *U*-test was used to compare their peer evaluations of the learning effects, problem-reasoning abilities, and teamwork. We used GraphPad Prizm 9.0 (La Jolla, CA) to conduct all statistical analyses, with significance established at *P* < 0.05.

## Results

### Improved performance based on higher scores in the FC-TBL (vs LBL) group

**Fig. 2:**
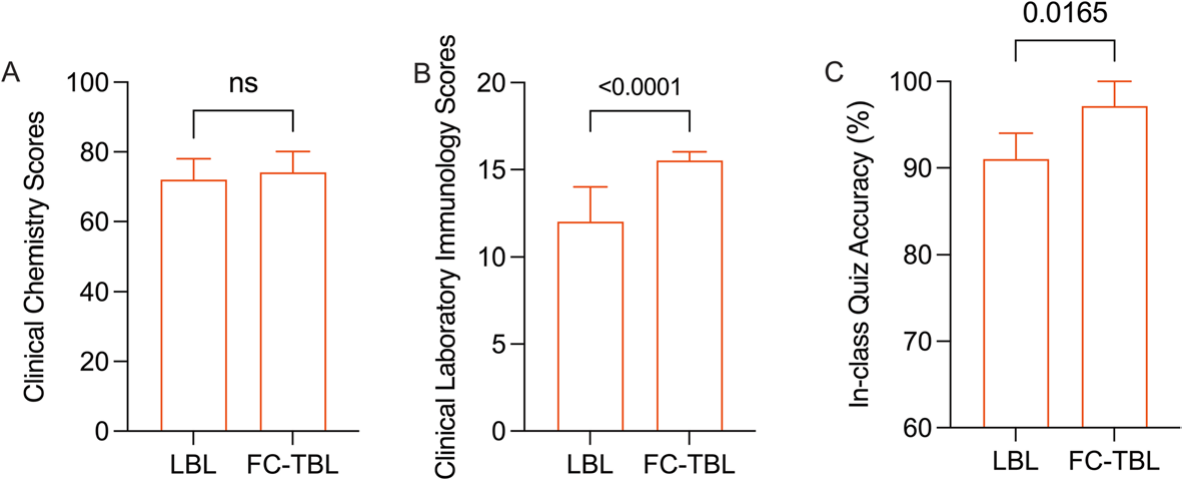
Comparison of in-class quiz and final exam results between the two study groups. The final examination scores of clinical chemistry were compared by an independent samples *t*-test, and the final examination scores of clinical chemistry as well as accuracy on the in-class quizzes were compared by Mann–Whitney *U*-test.**a** Comparison of mean clinical chemistry scores between LBL and FC-TBL groups. **b** Comparison of mean clinical laboratory immunology scores between LBL and FC-TBL groups. **c** Comparison of mean in-class quiz accuracy between LBL and FC-TBL groups. LBL: lecture-based learning, FC-TBL: flipped classroom with team-based learning. *P* value was shown in the figure. ns: no significant difference

We used the final examination scores from clinical chemistry as a baseline for both groups, which were taught with LBL. Here, there were no statistical intergroup differences (73.94 *vs* 74.84 *P* > 0.05, Fig. 2.A). Following the course proceedings, our results suggested that FC-TBL significantly improved academic performance throughout the learning process, with median final examination scores of 12 (IQR 12.00–14.00) and 15.5 (IQR 14.25–16.00) for the LBL and FC-TBL groups, respectively (*P* < 0.001, Fig. 2.B); moreover, there were significant differences in median accuracy on the in-class quizzes, at 91.00% (IQR 82.50%–93.75%) and 97.14% (IQR 94.29%–100%), respectively (*P* < 0.05) (Fig. 2C).

### FC-TBL was highly suitable for clinical laboratory immunology

All 62 participants completed the satisfaction survey, with a questionnaire return rate of 100%. We prepared a total of 14 questions to compare subjective responses between the FC-TBL and LBL groups. As shown in Table 2, the FC-TBL group reported significantly higher interest levels than the LBL group (*P* ≤ 0.001). Most of the FC-TBL group submitted positive “yes” responses to the following items: “should be used by more clinical caboratory immunology teachers”, “is a more scientific way for medical teaching”, “stimulates your interest in learning clinical laboratory immunology”,” strengthens your intrinsic motivation”, “develops your self-directed learning skills”, “improves your problem-solving skills”, “provides benefits in terms of long-term memory”, “helps you understand the course objectives”, “I can easily browse lessons according to my own situation any time”, “FC-TBL may not bring an increase in workload”, “The delivery of knowledge in an FC-TBL is fragmented and unsystematic”, “is feasible for the current educational environment”, “is an effective teaching model that is worthy of promotion”. On the whole, the students in the FC-TBL group could gain more solid knowledges by enhancing their learning interest and intrinsic motivation in the current educational environment. They also believed that FC-TBL was more suitable for use in clinical laboratory immunology and should be frequently employed by teachers. As to the question “helps you prepare for clinical laboratory immunology exams?”, 17 participants gave positive response compared to only 5 “yes” responses in LBL group (*P* = 0.001). Our results suggest that FC-TBL can facilitate the mastery of key knowledge points over limited periods of time.

**Table 2:** Participant reactions to the online FC-TBL teaching strategy (*n* = 62)

Items	Teaching strategies		Static
	FC-TBL (*n* = 31) (*n*, %)	LBL (*n* = 31) (*n*, %)	χ^2^ (*P*)
Should be used by more clinical laboratory immunology teachers?	26 (84%)	2(6%)	37.51(< 0.001)
Is a more scientific way for medical teaching?	24 (77%)	7(22%)	18.65(< 0.001)
Stimulates your interest in learning clinical laboratory immunology?	26 (84%)	5(16%)	28.45(< 0.001)
Strengthens your intrinsic motivation?	26 (84%)	4(12%)	31.26(< 0.001)
Develops your self-directed learning skills?	30 (97%)	6(19%)	38.15(< 0.001)
Improves your problem-solving skills?	27 (87%)	7(22%)	26.05(< 0.001)
Helps you prepare for clinical laboratory immunology exams?	17 (55%)	5(16%)	10.15(0.001)
Provides benefits in terms of long-term memory?	27 (87%)	2(6%)	40.49(< 0.001)
Helps you understand the course objectives?	28 (90%)	6(19%)	31.52(< 0.001)
I can easily browse lessons according to my own situation any time?	23 (74%)	9(29%)	12.66(0.001)
May not bring an additional increase in workload?	29(94%)	11(35%)	22.83(< 0.001)
The delivery of knowledge in an FC-TBL is fragmented and unsystematic?	25(81%)	4(12%)	28.57(< 0.001)
Is feasible for the current educational environment?	24 (77%)	3(9%)	28.93(< 0.001)
Is an effective teaching model that is worthy of promotion?	27 (87%)	2(6%)	40.49(< 0.001)
Summary (%)	25.6 (83%)	5.2 (17%)	376.9(< 0.001)

The two groups were compared using Pearson's chi-square test

## FC-TBL promoted learning interest and problem-solving abilities

We asked the FC-TBL group to subjectively compare the two teaching strategies by responding to eight items on the satisfaction survey (Table 3). As such, they reported several benefits: Compared to LBL, most believed that FC-TBL could significantly “Increase learning motivation,” “Promote self-directed learning skills,” “Extend more related knowledge,” “Enhance problem-solving abilities,” “Enhance clinical reasoning abilities,” and “Enhance communication skills” (*P* < 0.05). Thus, FC-TBL was considered a successful way to enhance problem-solving abilities by working with team partners. However, they did not report any differences for “Improve peer interaction” or “Help with memorizing basic knowledge” between approaches (*P* > 0.05). A potential reason for this may have been the lack of physical meetings during the pandemic.

**Table 3:** FC-TBL peer evaluations of the learning effects, problem-reasoning abilities, and teamwork (*n* = 31)

Items	LBL group (scores)	FC -TBL group (scores)	*P*
Increase learning motivation	4(3–5)	5(4–5)	0.024
Promote self-directed learning skills	4(3–5)	5(4–5)	0.024
Improve peer interaction	4(3–5)	5(4–5)	0.073
Extend more related knowledge	4(3–5)	5(4–5)	0.003
Help with memorizing basic knowledge	4(4–5)	4(4–5)	0.982
Enhance problem-solving abilities	4(3–5)	5(4–5)	0.000
Enhance clinical reasoning abilities	4(3–5)	5(4–5)	0.026
Enhance communication skills	4(3–5)	5(4–5)	0.007

Participants rated each statement on a 5-point scale, and scores were divided into 5 grades, ranging from 1 point (strongly disagree) to 5 points (strongly agree). We then calculated total scores. Data are shown as medians (25%-75%) and were analyzed via Mann–Whitney *U*-test

## Participants’ suggestions benefit for the further teaching of FC-TBL

All students (31/31, 100%) responded to the suggestion survey, and 48.38% (15/31) students held positive attitude to FC-TBL teaching strategy compared to 25.81% (8/31) students who considered FC-TBL teaching strategy still needs continuous improvement, and 25.81% (8/31) students reported that they believed FC-TBL teaching strategy was perfect and no further suggestions. Hence, we divided the suggestions into positive and improvement parts. As showed in Table 4, students’ suggestions mainly focused on the positive suggestions including frequency and interval time of FC-TBL as well as options and types of FC-TBL. They wished to increase more opportunity by more topics, class hours, forms in future class because it increased their interest in abstract knowledge while highlighting individual abilities and strengths through the teamwork process. Suggestions for improvement from 8 students including evaluation rules, participation equity as well as frequency and interval time of FC-TBL. They suggested that teachers should establish clear course evaluation rules, as many students failed to show their abilities or demonstrate proficiency on assignments due to limitations of preparing time, frequency and interval time; in this regard, the evaluation criteria should entail comprehensive scores that reflect the specific difficulty levels of each FC topic. Interestingly, we found “frequency and interval time” was the common suggestion in positive and improvement parts (8 vs 4), which promoted teachers to seriously think about teaching plan in future class with FC-TBL. We believe all these suggestions will benefit for the further teaching of FC-TBL for clinical laboratory medicine course.

**Table 4:** Participant suggestions for the FC-TBL teaching strategy

FC-TBL (*n*, %)	Examples from survey responses
Positive suggestions (15, 48.38%)	
Frequency and interval time (8, 25.81%)	a) Choosing one topic for each chapter to teach with TC-TBL b) Appropriately increasing the times and class hours of FC-TBL
Options or types of FC-TBL (5, 16.13%)	a) Expanding forms, and deadlines can be appropriately flexible b) Giving more available topics c) Adding some incentives for outstanding students
Learning effect (2, 6.45%)	Class is lively and interesting, and help to master the obscure knowledge
Suggestions for improvement (8, 25.81%)	
Evaluation Rules and Equity (4, 12.90%)	a) Not everyone has opportunity to show their talents due to the class time which affected the evaluation to the student b) Evaluation rules based on the difficulty of topics, students’ performance and cost time c) Give more students opportunities to show their talents
Frequency and interval time (4, 12.90%)	a) The time interval between flipped classrooms was too close b) FC-TBL takes not too long time in and after class

## Discussion

In China, continuous higher education reforms have influenced educators to adopt student-centered pedagogies over teacher-centered approaches. Teaching must change accordingly, as most traditional methods no longer meet the requirements of modern medical education [23, 24]. As a compulsory course for laboratory medicine students, clinical laboratory immunology emphasizes a combination of basic immunology theory and clinical practice. Many such courses are now taught through the FC approach, although its key theoretical points have emerged through years of learning theory research [25]. In the FC model, students are provided with information prior to class to promote better engagement through active learning during attendance [26–28]. Meanwhile, research has shown that the TBL approach enhances confidence in scientific reasoning, facilitates perusal of the literature, increases one’s understanding of more obscure textbook information, improves clinical and teamworking skills, and helps students develop the skill mastery needed to complete in-class and final examinations [29, 30].

In this study, our FC-TBL strategy was based on both previous research and practical experience with different teaching strategies, including a range of in-class interactions with students [31]. Based on our survey results, the participants observed flaws in traditional LBL, but had very positive perceptions of FC-TBL (Table 2). From the students’ perspective, the same contents were more attractive and interesting when taught via the FC-TBL strategy. In turn, they thoroughly memorized the information needed to prepare for their in-class presentations and discussions [31, 32]. Although they found that FC-TBL did not increase their workloads, they also obtained higher test scores than participants who received LBL instruction, that may be due to the active effect for the learning attitude, success, and positive behavior of students by FC-TBL teaching strategy [33].

For the FC-TBL strategy, we selected course contents that pertained to both professional knowledge and clinical practice. Here, the instructors provided easily comprehensible pre-class materials to help participants understand and master key points through self-study. Those who read the materials could discuss them in assigned teams, thus improving their problem-solving abilities while achieving our teaching goals [34]. These results are consistent with existing evidence that FC-TBL helps students develop creativity, collaboration, and cohesion [32, 35, 36]. However, we know of no other studies that have used FC-TBL to teach clinical laboratory immunology, especially given the unique course contents. Compared to traditional LBL, our results suggest that FC-TBL can significantly improve test scores (Fig. 2), and that it is both more suitable for mastering theoretical knowledge and strengthening the connection between education and clinical practice. This was associated with the interaction between students, resulting to more knowledge gained by the disadvantaged participants [6]. Hence, students will benefit from the FC-TBL teaching strategy characteristic by active learning and interaction with each other, perfectly matched in aspect of constructivism [37].These findings support a range of studies showing that FC-TBL is a highly effective learning method.

After surveying participants to clarify their perceptions of the two teaching strategies, we found that they were highly accepting of FC-TBL overall, which is also consistent with previous research [38–40]. FC-TBL significantly increased learning motivation, which encouraged participants to expand their knowledge through self-study efforts and communicating with team members (Table 3). At the same time, they gained problem-solving and clinical reasoning abilities. However, the participants did not report improvements in peer interaction or basic knowledge memorization, which may be because they were unable to personally meet and lacked a physical school learning environment due to COVID-19 restrictions. Finally, most students held positive attitudes towards FC-TBL teaching strategy, and expressed the desire for increased frequencies and more options or types of FC-TBL, which may have stemmed from the relatively few courses’ offerings during the semester in which this study was conducted (Table 4). In this regard, they wanted more opportunities to demonstrate their abilities based on fairer evaluation criteria. Each student would actively study in the group if the reward was given only for one work or student, and team enjoyment and motivation variables has effect on learning effects [41, 42]. Hence, we will focus on the fairness of rewards and optimize the frequency of FC-TBL classes to motivate each student to study in the future.

## Limitations

This study also had some limitations, especially due to the COVID-19 pandemic. First, there were restrictions on the available time, course contents, and pre-class materials used in the FC-TBL component, all of which can be optimized in future applications. Second, a one-year interval separated our engagement with the two study groups. However, we do not believe this affected the results because we normalized our intergroup comparison by referring to the final scores on clinical chemistry in each case. Third, all participants were third-year Medical Laboratory students who were taking clinical laboratory immunology, which limits generalizability to other courses and majors. Finally, the study sample was relatively limited in number. Future studies can improve accuracy and robustness by recruiting a larger number of participants.

## Conclusions

Our online FC-TBL strategy aimed to increase understanding, enhance reasoning, and improve the application of basic knowledge by providing students with pre-class materials, facilitating knowledge integration, and offering a team-based discussion format, all of which are essential in future clinical practice. Based on our subjective and objective assessments, we found that the students by FC-TBL teaching showed more problem-solving abilities and mastering theoretical knowledge in relate to strengthening the connection between education and clinical practice compared to LBL, which indicated FC-TBL was highly suitable for use in clinical laboratory immunology. In general, online FC-TBL is an effective way to promote learning, especially under the constraints of the ongoing COVID-19 pandemic.

## Data Availability

Data collection containing teaching data is available upon request from the corresponding author.

## Abbreviations

(COVID-19): Coronavirus Disease 2019
(FC): Flipped Classroom
(FC-TBL): FC with TBL
(LBL): Lecture-Based Learning
(TBL): Team-Based Learning
